# Notes and News

**Published:** 1890-09-06

**Authors:** 


### Notes and News.

Collected and Communicated.

Pontefract kept Hospital Sunday in a most melodious manner with an outdoor concert. The Mayor graced the proceedings by his presence, and the weather being fine the audience numbered about 4,000. Several well-known hymns were included in the programme, but also some difficult choruses from the "Messiah" and "Judas."

Sir Thomas Crawford delivered the presidential address at the Sanitary Congress. He said that a cynical world was prone to regard man as a chattel, the care of which was monopolised by the three faculties—the clergy for his soul, the doctors for his body, and the lawyers for his worldly possessions. Of the three the lawyers seemed to be the most successful, for while wealth increased by leaps and bounds, the moral and physical condition of the people lagged sadly behind. The remedy lay in united and well-directed efforts on the part of the learned professions in the cause of public health, and towards creating a healthy public opinion on the importance of sanitary legislation, and the due administration of all laws bearing upon the health and physical condition of the people, and more especially of the working classes.

It is impossible to note all the interesting papers read at the highly successful meeting of the Sanitary Congress last week, but from a popular point of view a very interesting one was by Mr. W. Jago, on "Sanitation in Breadmaking," in which he laid down the contention that bread should be of the best possible composition, must be made by the best processes, and with absolute cleanliness. Among the processes used in the manufacture of bread, fermentation was far superior to any other mode of aëration; the aëration of bread by yeast giving a characteristic pleasant flavour, and being in itself a safeguard against the use of unsound flours. Most crying among all the evils of ordinary bread-making was that of dough being kneaded by hand, and the public should insist on the adoption of mechanical appliances by the baker. Bread should be baked in ovens free from ashes and smoke, and then allowed to cool in a special room of moderate temperature. Some of the meetings were simply crowded, and the audience was always enthusiastic and intelligent.

Mr. George A. Humble, M.D., M.R.C.P. Lond., has been appointed Consulting Physician to the General Hospital of the Rio Negro, Patagones, Argentine Republic, South America.

The annual report of the Miller Hospital and Royal Kent Dispensary tells of 187 in-patients and 1,682 accidents attended. The branch at Lee is now in full working order. The conclusion of the report gives the old story: "From the balance-sheet it will be seen that an exceptional sum of no less than £1,309 9s. 9d. received as legacies during 1889-90, had to be utilised towards meeting the current expenditure, and that notwithstanding this a balance of £246 2s. 11d. was due to the bank on March 31st last, the committee feel, therefore, compelled to draw the especial attention of the governors to the fact that the expenditure, despite the most rigid economy, is largely exceeding the annual income, and that most strenuous efforts must now be made to raise a further sum of, at least, £1,000 per annum, to obviate the necessity for the selling of the funded monies, and thereby reducing the income to meet recurring deficiencies."

The Marquis of Hertford laid the stone of the new Birmingham Ear and Throat Hospital on August 26th. Lord Leigh presented a silver trowel to the Marquis, and read an address detailing the history of the old institution. The proceedings passed off well, Lord Hertford making a short but appropriate speech. The building is to be of the style of architecture known as Queen Anne's, and will be built of red brick with terra cotta dressings, and roofed with red tiles. It will consist of five storeys. The ground floor is to be used by the out-patients to the number of 200, and will contain rooms for the medical officers, the dispenser, and the secretary. There will also be a strong room, and a mortuary—the latter approached from the outside. The first floor will contain two day-rooms, two meal-rooms, operating-room, ward, matron's apartments, baths, with all modern sanitary appliances. The second floor will contain three wards, house surgeon's apartments, nurses' room, linen-room, and baths, and all the necessary sanitary arrangements. The third floor will contain kitchen, scullery, and four bed-rooms. There will be accommodation for twenty in-patients. The building will have all the modern improvements and requisites that are necessary for a new special hospital.

A new Medical Practitioners Bill has been introduced to the Victoria Legislative Assembly by Mr. Deakin. It is to be known as the Medical Act 1890, and is divided into four parts, as follows:—Part 1, the Medical Council of Victoria; part 2, legally qualified medical practitioners; part 3, medical witnesses; and part 4, schools of anatomy. The bill abolishes the Medical Board of Victoria, and creates in its stead a council, to be styled the Medical Council of Victoria. The council is to consist of fifteen members. Of these, four are to be appointed by the Governor in Council, ten to be elected by the registered medical practitioners resident in Victoria, and one to be elected by the senate of the University of Melbourne. The members are to hold office for a term of five years, and the council is to elect its own president. Provision is made for the registration of duly qualified practitioners. In the third schedule to the Bill a list is provided of the recognised universities, colleges, and bodies, and any person possessing the qualifications of such bodies may be registered. The council is empowered to add to this list or remove from it any names, or refuse to register any qualifications of any body whose course of study or examinations of students, in the opinion of the council, have become insufficient to secure a proper amount of skill and knowledge. The council has power to erase from the list the name of any medical practitioner convicted of felony or misdemeanor, or who has in consequence of any misconduct, professional or otherwise, been erased from the roll or list of graduates of any recognised body.

THE following vacancies are announced this week, the amount of the salary in each case being given after the name of the institution:—

**Resident Medical Officers.**

St. Peter's Hospital, Henrietta Street, House Surgeon—£95, board, etc.

Chelsea Dispensary, Sloane Square, House Surgeon and Secretary —£95, rooms, etc.

Taunton Hospital, House Surgeon—£100, board, etc.

Worcester County Asylum, Third Assistant Medical Officer—£100, board, etc.

Bury St. Edmund's Hospital, House Surgeon—£80, rooms, etc.

Crichton Institution, Dumfries, Junior Medical Assistant—£100.

Salford Royal Hospital, House Surgeon—£100, board, etc.

**Non-Resident Medical Officers.**

Owens College, Manchester, Junior Demonstrator in Physiology and Histology—£100.

Queen's College, Birmingham, Tutor in Midwifery and Gynæcology.

The Hospital for Women, Soho Square, Clinical Assistants.

North-West London Hospital, Assistant Physician.

---

THERE is some little ill-feeling at Bury St. Edmunds at the unexpected appearance of an advertisement for a new house surgeon. The present officer has been true to his post for about 30 years, and has managed to win much affection and respect. It is not from him that the complaints arise, but the public having aroused a discussion on the subject, it would be well for the committee to fully explain their proceedings.

---

THE enormous work done by our London dispensaries is scarcely taken into proper account by the public. Now, here is the Chelsea, Brompton, and Belgrave Dispensary, with a total of 17,842 attendances and 5,764 visits for one year. This is only the dispensary, and what is more, it is not a "provident" but a gratuitous one, and, therefore, not so greatly in favour with the modern hard-headed philanthropist. But it records a fair income, with help from the clergy and the Hospital Saturday and Sunday Funds. It has been in existence since 1812, and shows signs of continuing for another 70 years, though very few Londoners do know of its work and ways.

---

GREAT indignation having been raised in Melbourne by Mr. Fitzgerald's (once of the Mercer's Hospital, Dublin) evidence against the Melbourne Hospital, Mr. Girdlestone, Dr. Grant, and Dr. Moore have come forward and corroborated it. The place is a poison-nest, according to Mr. Fitzgerald, and ought to be burnt; patients, physicians, and nurses alike die off within its walls. Dr. Youl, the city coroner, also gave some extraordinary evidence. He said that some of the bricks in the hospital walls were so porous that with the aid of a blow-pipe he could blow a candle out on the other side of the wall. The walls were just of such a character as to receive poisonous germs and to keep the hospital in a continuously septic condition. Notwithstanding all the facts that were elicited at the inquiry which was held, the allegations which he made were discredited; but a number of his suggestions had since been adopted. Mr. Fitzgerald did not agree with his views at first, but he had been since converted. Notwithstanding the improvements which had been effected, the hospital was anything but what it ought to be. Beds were never allowed to be vacant for more than a few hours, so that they never had a rest, and this practice was greatly against the recovery of patients. Patients succeeded one another so quickly that in one week he had held inquests on three bodies taken one after another from the same bed. No doubt the patients heard of the fate of their predecessors, and that did them no good. The management of the hospital was costly, patients requiring more stimulants than usual, owing to the impurity of the air. Every attempt seemed to be made to keep it clean, but it was impossible to make it suitable for its purpose owing to its bad construction. The death-rate of the hospital was 17 per cent., as against 13 per cent. in the Alfred Hospital, and 11 per cent. in the Prince Alfred Hospital, Sydney.

